# Developmental Changes in Learning: Computational Mechanisms and Social Influences

**DOI:** 10.3389/fpsyg.2017.02048

**Published:** 2017-11-23

**Authors:** Florian Bolenz, Andrea M. F. Reiter, Ben Eppinger

**Affiliations:** ^1^Chair of Lifespan Developmental Neuroscience, Department of Psychology, Technische Universität Dresden, Dresden, Germany; ^2^Department of Neurology, Max-Planck-Institute for Human Cognitive and Brain Sciences, Leipzig, Germany; ^3^Department of Psychology, Concordia University, Montreal, QC, Canada; ^4^PERFORM Centre, Concordia University, Montreal, QC, Canada

**Keywords:** reinforcement learning, cognitive modeling, decision-making, social cognition, lifespan, developmental neuroscience

## Abstract

Our ability to learn from the outcomes of our actions and to adapt our decisions accordingly changes over the course of the human lifespan. In recent years, there has been an increasing interest in using computational models to understand developmental changes in learning and decision-making. Moreover, extensions of these models are currently applied to study socio-emotional influences on learning in different age groups, a topic that is of great relevance for applications in education and health psychology. In this article, we aim to provide an introduction to basic ideas underlying computational models of reinforcement learning and focus on parameters and model variants that might be of interest to developmental scientists. We then highlight recent attempts to use reinforcement learning models to study the influence of social information on learning across development. The aim of this review is to illustrate how computational models can be applied in developmental science, what they can add to our understanding of developmental mechanisms and how they can be used to bridge the gap between psychological and neurobiological theories of development.

In our daily lives, we constantly need to learn about the conditions of our environment to improve our future choices. Which ice cream will I enjoy most? What school should I send my children to? How much money should I save for retirement? While the kinds of choices we have to deal with change throughout the lifespan, so do the strategies with which we approach decisions in order to find an optimal solution. Understanding how learning and decision-making is affected by age is therefore crucial for adapting structures and processes in educational, occupational, or health contexts to different target groups.

Over the past few years, computational approaches such as reinforcement learning (RL) models (Sutton and Barto, 1998) have become increasingly popular in psychology and cognitive neuroscience. One big advantage of these models over descriptive (verbal) theories is that they allow us to explicitly formalize cognitive processes. That is, we can use these models to make explicit numerical predictions regarding the effects of experimental manipulations on outcome measures. This level of specificity is difficult to achieve with verbal theories alone. Moreover, computational models can be used to simulate behavior and thus also to simulate potential limitations of sub-processes that come with development, aging, or pathology (e.g., Nassar et al., 2016). Another important qualitative advantage of computational models is that they can provide access to latent cognitive processes. For example, many researchers are interested in the psychological processes underlying performance monitoring or in the question of how people handle conflicts between habitual and goal-directed response tendencies. Computational models allow us to formalize these latent processes and make them accessible for empirical approaches. Finally, one of the main reasons for the increasing popularity of these models in cognitive neuroscience is that they can be used to derive time varying variables of computational processes that can be correlated with neurophysiological data (e.g., O'Doherty et al., 2004; Gläscher et al., 2010). These so-called “model-based” analyses can provide insights into the neural dynamics underlying cognitive processes that are difficult to achieve with the standard approaches.

For researchers in lifespan developmental neuroscience, computational modeling techniques are particularly promising because they might provide new insights into developmental processes that lead to changes in learning and decision-making and how they relate to the development of neurobiological function (van den Bos et al., 2017). While most of the current research in the area of computational neuroscience has focused on individual learning and decision-making, there is an increasing interest in using computational methods to understand social influences on learning and choice behavior (Behrens et al., 2008, 2009; Diaconescu et al., 2014, 2017). This new emerging research trend seems particularly relevant for the developmental field because of the immense impact of social influences on developmental processes, especially during childhood and adolescence (Ainsworth, 1989; Herrmann et al., 2007; Blakemore, 2008; Somerville, 2013).

In this review, we will outline how developmental psychologists can make use of modeling approaches to study age-related changes in learning and decision-making. We will demonstrate how the basic computational algorithms of RL can be extended and modified to address questions about the developmental trajectories of learning and decision-making processes across one's lifespan. The first section will introduce and briefly summarize the fundamental principles of RL models. We will then discuss how the computational level is linked to psychological constructs and theories about human development by giving examples from the literature on the development of learning and decision-making in non-social settings. Finally, the employment of modeling techniques in the context of social decision-making will be reviewed, and we will show how existing models can be applied to developmental questions on social learning.

## Basic reinforcement learning models

Many of our preferences (e.g., for one type of ice cream over another) are shaped by experience-driven learning mechanisms. That is, we sample our environment (our favorite ice cream parlor), and depending on our evaluation of the outcomes, we update the value representations of the different types of ice cream.

**Figure 1:**
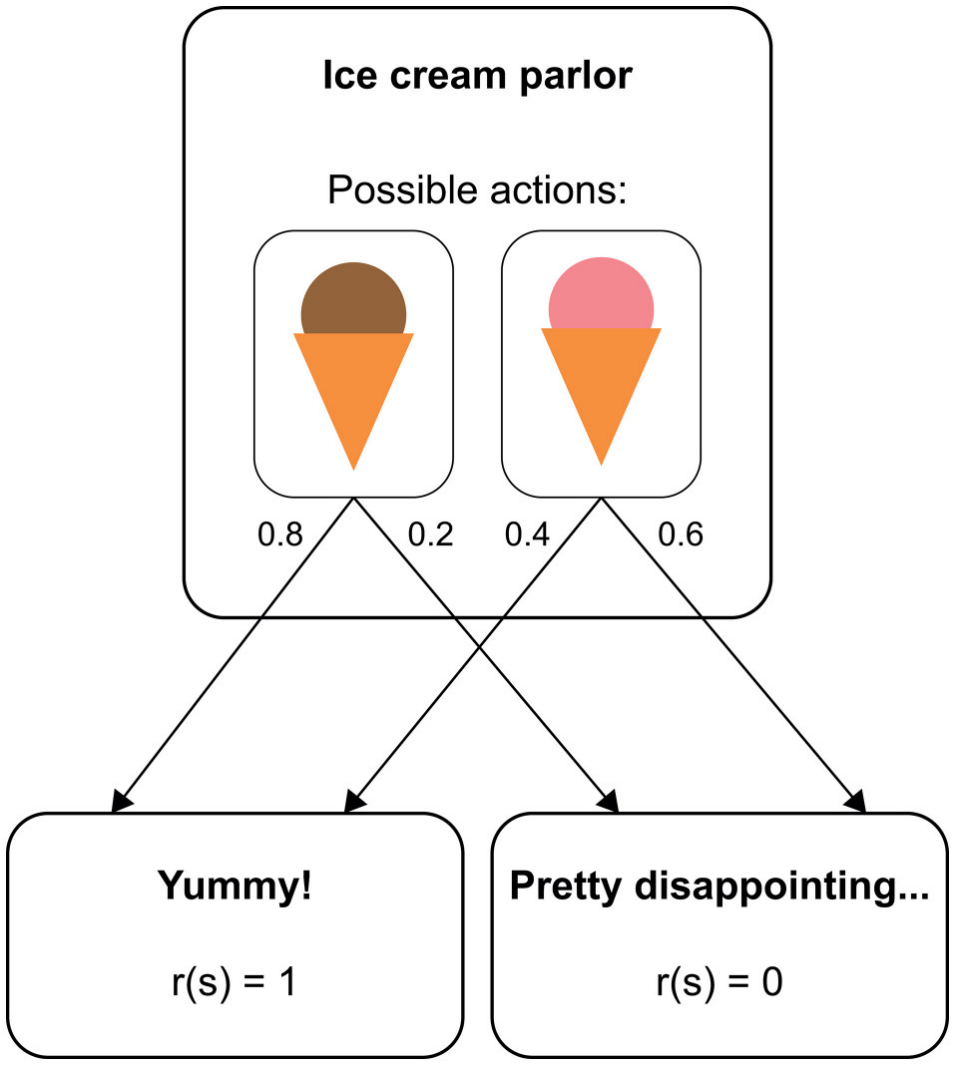
Structure of a real-world decision as a Markov decision process. The state “ice cream 922 parlor” has two available actions, “chocolate ice cream” and “strawberry ice cream”. With a certain probability (represented by numbers next to the arrows), each choice leads to either a reward state or a non-reward state.

RL models provide a formalization of how a human (or non-human) agent learns from experience to maximize her reward in a given environment (Sutton and Barto, 1998). In many situations, RL can be understood in terms of a *Markov decision process (MDP)*. An MDP consists of distinct states an agent can find herself in (e.g., different sensory inputs), and each state provides the agent with a set of available actions. On performing one of these actions, the agent moves to a new state according to a transition function that defines the probability of arriving in this state given the previous state and the selected action. Figure 1 illustrates how this structure applies to a simplified example (similar to a two-alternative forced choice task in a psychological experiment). Here, a person repeatedly chooses between two types of ice cream, where each choice is followed by either a pleasant taste (outcome value = 1) or a neutral taste (outcome value = 0). The outcome values are on an arbitrary scale and only become meaningful in relation to each other. Typically, positive values represent rewards and negative values losses or punishments. Importantly, whether a type of ice cream is experienced as pleasant or not can be different even after identical choices (maybe because there is variance in the product quality or because the taste also depends on factors that we do not account for, such as the person's mood). If the probability of experiencing a pleasant taste is higher after choosing one type of ice cream than after choosing the other, the person will eventually develop a preference for the most pleasant type of ice cream, thus maximizing the number of pleasant taste experiences in the long run.

In order to optimize behavior, the agent needs to iteratively update her reward expectation of different actions. In RL models, this is formalized as a state-action value *Q(s,a)* that represents the subjective expectation of reward from performing action *a* in state *s*. The example introduced above involves two state-action values, Q(ice cream parlor, chocolate) and Q(ice cream parlor, strawberry). If both state-action values are equal, the participant does not prefer either type of ice cream. With learning, the participant continuously updates her prediction about the value of the two flavors based on the perceived discrepancy between the expected and the actually experienced reward. This discrepancy is expressed by the *reward prediction error* δ that is computed after an agent has performed action *a* in state *s*:

(1) $$\begin{array}{c} \delta =\underset{\text{experienced reward}}{\underset{︸}{r\left(s^{\prime }\right)+Q\left(s^{\prime },a^{\prime }\right)}}-\underset{\text{expected reward}}{\underset{︸}{Q\left(s,a\right)}} \end{array}$$

Here, *s*′ is the new state the agent has moved to, where she receives a reward with value *r*(*s*′) and is going to perform another action *a*′. Importantly, in this equation the experienced reward is the sum of the immediately obtained reward *r*(*s*′) and the prospective future reward represented by the state-action value for the action in the new state *Q*(*s*′, *a*′).

To illustrate how the computation of a reward prediction error works, consider a person who expects chocolate ice cream to be moderately rewarding (Q(ice cream parlor, chocolate) = 0.6) and, having selected chocolate ice cream, experiences a pleasant taste (*r*(*s*′) = 1). In this example task, the experienced reward is completely determined by the immediate reward because after tasting the ice cream, no further actions are available (*Q*(*s*′, *a*′) = 0). Then, the reward prediction error is computed as the difference between experienced and expected reward, which is δ = (1+0)−0.6 = 0.4. If the experienced reward is larger than the expected reward (better than expected outcome), the reward prediction error δ will take a positive value. Conversely, if the experienced reward is smaller than the expected reward (worse than expected outcome), δ will be negative.

The reward prediction error is then used to update the state-action value:

(2) $$\begin{array}{c} Q(s,a)\leftarrow Q(s,a)+\alpha \delta \end{array}$$

**Figure 2:**
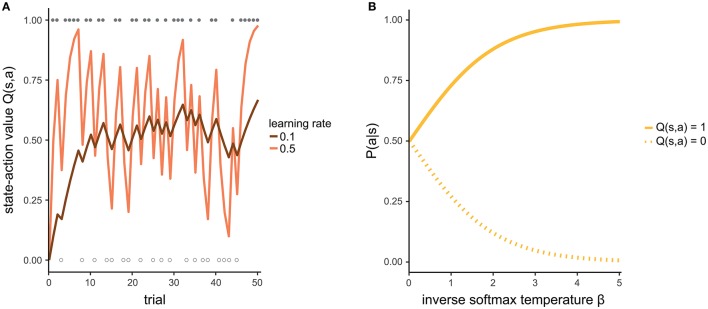
**(A)** Development of a state-action value for two different learning rates. For the purpose of illustration, we assume that the agent makes identical choices across all trials. Filled and empty circles indicate trials in which the action was rewarded (*r* = 1) or not rewarded (*r* = 0), respectively. With a high learning rate (light line), the state-action value estimate fluctuates strongly, representing the rewards of the most recent trials. In contrast, with a low learning rate (dark line), the state-action value is more stable because it pools over more of the previous trials. **(B)** The higher the inverse softmax temperature, the more it is likely to prefer an action with a state-action value of 1 over another action with a state-action value of 0.

The individual learning rate α indicates how strongly the most recent experience is weighted relative to previous experiences when updating the state-action value. If α = 0, the new experience is not at all taken into account (even if your current chocolate ice cream does not taste as predicted, you will not change your future expectation) and the state-action value remains unchanged. If α = 1, the new state-action value is completely updated by the new experience (your attitude toward a type of ice cream is determined only by the last time you tasted it). Intermediate values of α reflect a certain balance between the recent experience and previous ones. That is, you consider both your last cup of ice cream as well as other ice cream you have had in the past when making a decision. Figure 2A illustrates how a state-action value evolves given different learning rates.

### From learning to action selection

So far, we have only considered the question of how value expectations are built and updated. However, for an agent it is also important how to translate value representations into actions. That is, how does our preference for one type of ice cream over the other makes us choose one of them? Most RL models assume that actions are selected probabilistically. The probability that an agent selects a particular action *a*_*i*_ out of all available actions is usually modeled with a softmax function:

(3) $$\begin{array}{c} P(a_{i}|s)=\;\frac{\exp(\beta Q(s,a_{i}))}{\sum_{k}\exp(\beta Q(s,a_{k}))} \end{array}$$

The inverse softmax temperature β controls the extent to which differences in state-action values affect action selection. If β = 0, differences in state-action values have no effect and all actions are selected with equal probability. With increasing values of β, it becomes more and more likely to select the action with the highest state-action value. The specific form of the softmax function guarantees that the probability of selecting an action is relative to the respective state-action value and that the probabilities of all available actions in one state add up to 1. Figure 2B exemplifies how β affects the mapping from state-action values to probabilities.

## Linking model parameters to psychological constructs

### Learning rate

#### Learning from positive vs. negative prediction errors

Until now, we have treated positive and negative prediction errors alike in terms of their effects on updating value representations. However, it is intuitively clear that a negative prediction error might have different implications for behavior than a positive prediction error. For example, your favorite ice cream might suddenly induce an allergic reaction (negative prediction error). Consequently, you will shift your preference and avoid this ice cream. In contrast, in most cases, positive prediction errors will reinforce existing behavior or preferences. Psychological theories assume that the ability to adapt behavior to negative consequence involves performance monitoring processes that rely on prefrontal structures such as the medial prefrontal and dorsolateral prefrontal cortex and the insula (Holroyd and Coles, 2002; Ullsperger and von Cramon, 2003; Ridderinkhof et al., 2004). In contrast, learning from positive outcomes has been suggested to involve dopaminergic projections to limbic and paralimbic areas such as the ventral striatum and ventromedial prefrontal cortex (vmPFC) (Schultz et al., 1997; Montague et al., 2004; D'Ardenne et al., 2008), although some subregions of the vmPFC may also be involved in error monitoring (Maier et al., 2015; Buzzell et al., 2017). Interestingly, these distinct neural systems have different developmental trajectories. Structural as well as functional magnetic resonance imaging (fMRI) revealed that prefrontal areas involved in learning from negative outcomes show a protracted development compared to limbic and paralimbic circuits involved in learning from reward (Sowell et al., 2003; Gogtay et al., 2004). Note however, that different subregions of the vmPFC show heterogeneous developmental trajectories (e.g., Shaw et al., 2008), which calls for a closer investigation of age-related effects in these areas. Moreover, a recent source localization EEG study (Buzzell et al., 2017) investigating error processing in 9–35 year old participants showed a linear association between age and error-related electrophysiological activity presumably originating from the insula or the inferior frontal gyrus. Both regions have been previously implicated in punishment-based learning (Palminteri et al., 2012) or the inhibition of maladaptive actions (Aron et al., 2004).

Consistent with the neuroimaging evidence for differential developmental trajectories in the brain systems involved in learning from positive and negative outcomes behavioral and electrophysiological studies indicate that children have problems in evaluating the informativeness of negative feedback during probabilistic learning (Crone et al., 2004; van Duijvenvoorde et al., 2008; Eppinger et al., 2009). What has been missing in many of the previous studies on learning from positive vs. negative outcomes is a clear characterization of the computational mechanisms underlying each of these different behavioral strategies.

Standard RL models assume one common updating mechanism for all kinds of outcomes regardless of their valence, which makes it difficult to capture diverging developmental trajectories for learning from gains vs. losses. To overcome this limitation, researchers have extended classical RL models by introducing two distinct learning rates, α_+_ and α_−_, instead of a single learning rate parameter. Thus, the specific learning rate can be applied depending on whether the reward prediction error signals a better than expected or a worse than expected outcome.

(4) $$\begin{array}{c} Q\left(s,a\right)\leftarrow \{\begin{array}{c} Q\left(s,a\right)+\alpha_{+}\delta \;if\;\delta \geq 0\\ Q\left(s,a\right)+\alpha_{-}\delta \;if\;\delta <0 \end{array} \end{array}$$

The only study that has investigated learning from gains and losses across development showed that the impact of negative reward prediction errors decreased with age (van den Bos et al., 2012). Studies in adolescents found an age-related enhancement in the sensitivity to worse than expected outcomes from adolescence to adulthood (Christakou et al., 2013; Hauser et al., 2015). Taken together, the few existing studies point to substantial changes in the impact of gains and losses across childhood development and adolescence. Clearly, more research is needed in this area, and future studies should adjust the tasks and procedures in a way that younger individuals (i.e., younger than 8 years of age) can be included.

#### Adaptive learning rates

While distinct learning rates for positive and negative prediction errors allow for a somewhat more flexible responses to different situational demands, the impact of new information in these models remains constant over time. This is a questionable assumption, because most of our environments are changing dynamically and we have to flexibly adjust the degree of learning. For example, if you know that the quality of your favorite ice cream varies a lot between single scoops, it makes no sense to completely revise your value prediction just because the ice cream did not meet your expectations once. In contrast, if you learned that the ice cream manufacturer recently changed the recipe, even a single disappointing experience might make you buy a different type of ice cream next time.

The idea to adaptively adjust learning rates has also been implemented in RL models (Krugel et al., 2009). Here, the learning rate itself is updated in every trial depending on whether recent reward prediction errors increase (a change to the environment has occurred and the impact of new information should be high) or decrease (indicating stable environmental conditions). Recent work in adolescents shows no difference in learning rate adaptation between teenagers and adults in a reversal learning task (Javadi et al., 2014). However, so far, this is the only study in this domain, and childhood developmental differences have not (yet) been addressed. In addition, it should be noted that in current RL approaches the mechanisms that regulate the relationship between prediction error and learning rate are unclear. Recent work using Bayesian models of belief updating tried to address these shortcomings. For example, research by Nassar et al. (2016) on aging-related changes in learning rate adjustments showed that older adults have a specific deficit in uncertainty-driven learning that manifests as a problem in adjusting learning rates to small changes in prediction errors.

Future work should focus on developmental changes in the factors that regulate the degree of learning in dynamically changing environments. Furthermore, more research should be devoted to developmental differences in the interplay of medial prefrontal systems involved in the regulation of learning rates and their interaction with neuro-modulatory systems such as the norepinephrine and dopamine system. Finally, developmental disorders such as autism and attention deficit hyperactivity disorder are interesting research targets. For example, recent work suggests that adults with autism tend to overestimate the volatility of their environment, which makes them less sensitive to surprising environmental changes (Lawson et al., 2017). However, the ontogenetic development of these biases or their relationship to changes in neuro-modulatory systems remains to be determined.

### Softmax temperature

We sometimes deviate from what would be the optimal behavior in a given situation. In the framework of RL, this means that an agent might pick an option that does not have the highest state-action value among all available actions. The degree to which state-action values guide action selection is regulated by the inverse softmax temperature, where lower values indicate a high level of behavioral stochasticity (i.e., choices are barely controlled by state-action values and are mainly due to chance) and action selection becomes more and more deterministic with increasing parameter values.

Two major psychological interpretations of the softmax temperature parameter have been offered: On the one hand, one can think of it as representing the individual sensitivity to differences in state-action values. From this perspective, deviations from the most rewarding options are considered as being due to random noise in the selection process or the lack of distinct value representations for alternative actions. Thus, theories that connect lifespan developmental differences in the dopaminergic system with transformations in the neural signal-to-noise ratio (Li et al., 2010; Li and Rieckmann, 2014) make the prediction that this parameter is likely to change across development.

Alternatively, choice stochasticity can be regarded as reflecting exploration of one's environment (as opposed to exploitation of familiar options). The inverse softmax temperature parameter then represents the degree of exploratory behavior (e.g., Daw et al., 2006). Enhanced exploratory behavior and risk-seeing is thought to be characteristic of adolescent behavior (Crone and Dahl, 2012; Crone and Steinbeis, 2017), which leads to the hypothesis that differences in the inverse softmax temperature parameter could also be accounted for by age-related shifts with respect to exploratory tendencies.

Indeed, developmental studies find that adolescents' behavior is best described by RL models with higher stochasticity as compared to younger adults. Javadi et al. (2014) used a reversal learning task with adolescents and younger adults and found that adolescents' choices were less controlled by differences in reward expectations than the choices of younger adults. Likewise, Christakou et al. (2013) report a lower inverse softmax temperature in adolescents than in adults for behavior in the Iowa Gambling Task. These age differences in choice stochasticity were associated with task performance and can be interpreted as reflecting increased exploratory behavior in adolescence.

It is beyond the scope of both conventional RL models and decision-making tasks to make a clear statement on whether observed choice stochasticity represents deliberately exploratory or merely random behavior. For future research, it would be desirable to better differentiate how these two processes develop across the human lifespan. In order to disentangle exploratory from random behavior, Wilson et al. (2014a) have put forward a decision-making task where they manipulate the amount of information an agent has about the outcomes of the available options before making choices. The authors argue that higher preferences for a less-known option signal that a decision-maker assigns value to collecting information about the environment, which is indicative of exploratory behavior. They also formalize this idea in a computational model of the action selection process. A recent study by Somerville et al. (2017) applied this paradigm and found that strategic exploratory behavior increased from adolescence to adulthood, while random behavior remained constant.

### Model-based learning

The RL models formalized above represent *model-free RL* approaches. That is, the learner is not assumed to have explicit knowledge about the task structure (i.e., a model of the environment). Model-free RL is neither computationally nor cognitively very demanding because at decision time, one just needs to retrieve and compare a limited set of state-action values (Daw et al., 2005). However, state-action values can be adapted only retrospectively after an outcome has been experienced, and thus model-free RL can be rather slow and inflexible in dynamic environments. For example, having eaten chocolate cakes ad nauseam every day of last week, one might have a temporarily reduced preference for everything that tastes like chocolate. Nevertheless, a model-free learner would not be able to consider this devaluation of chocolate ice cream beforehand but would need to experience the new dislike of chocolate ice cream multiple times, until the respective state-action values are sufficiently updated.

An alternative RL approach, *model-based learning*, might be more appropriate to describe human behavior in such situations. Here, the agent is aware of the principles of the environment, for example the rewards associated with each state and the probabilities for moving between states, and can use this knowledge for forward planning. In the example above, anticipating the taste of chocolate, the model-based learner can immediately reduce the reward expectation of going for chocolate ice cream. While this allows for more flexible behavior, it comes at the cost of greater computational or cognitive effort.

Current psychological theories assume that human behavior is best described as a mixture of model-free and model-based RL strategies (Gläscher et al., 2010; Daw et al., 2011). By using a hybrid model of model-free and model-based RL and estimating the relative weight of both processes, several studies have investigated how model-based control develops across the lifespan. From childhood to early adulthood, the ability to make use of model-based RL strategies in a prominent decision-making task increases progressively (Decker et al., 2016; Li and Eppinger, 2016; Potter et al., 2017), and this effect is mediated by an increasing ability in fluid reasoning (Potter et al., 2017). In older age, the use of model-based strategies declines (Eppinger et al., 2013b; Worthy et al., 2014), a process that does not seem to be fully explained by age-related impairment in working memory capacity (Eppinger et al., 2013b). Thus, the development of model-based decision-making across the human lifespan parallels age-related differences in cognitive control that show maturation until adulthood and a decline with aging (Braver and Barch, 2002; Luna et al., 2015).

While developmental studies so far have focused on differences in model-based control between individuals, there is also an increasing interest in the intraindividual adaptation of the model-based weight. Research with young adults has shown that humans adapt the balance between model-free and model-based RL according to situational demands such as current working memory load (Otto et al., 2013a), stress (Otto et al., 2013b; Radenbach et al., 2015), predictability of the environment (Daw et al., 2005; Lee et al., 2014; Eppinger et al., 2017), or incentive size (Kool et al., 2017). How exactly the brain determines which of the two systems is in charge at one point in time and how this arbitration process is affected by age is an interesting avenue for future research. Moreover, there are also other important questions with respect to model-based processes and how they change with age that have not yet been addressed: For example, it is currently unclear how models of the environment are represented in the brain and how these representations are updated. The few available studies suggest that fronto-parietal networks are involved in model-based learning (Gläscher et al., 2010) and that the orbitofrontal cortex may play a role in presenting latent (not directly observable) information about the structure of the world (Wilson et al., 2014b; Schuck et al., 2016). However, we are far away from a clear characterization of the underlying neural processes and how they change as a function of development.

## Modeling analyses beyond parameter estimation

### Model selection

In the previous sections, we described how estimating the model parameters that best describe some behavior is an efficient way of specifying differences in learning and decision-making between age groups. However, the advantages of computational modeling are not limited to parameter estimation. Sometimes, there are multiple models available for explaining behavior in a task, each representing a different assumption about the involvement and interaction of specific cognitive processes. In this case, the direct comparison of competing computational models can reveal which out of a set of candidate models accounts best for behavior and can therefore be highly informative with respect to qualitative differences in cognitive processing between age groups.

For example, Palminteri et al. (2016) fitted RL models of increasing complexity to the behavior of adolescents and young adults in a probabilistic choice task. Specifically, these models differed with respect to whether they allowed for learning from counterfactual information (that is, information about the potential outcome of the option that was not chosen) and for the contextualization of outcomes relative to a reference point. They found that the simplest model explained adolescents' choices best while the most complex model accounted best for the decisions of the young adults. Thus, their findings suggest that learning strategies become more sophisticated with development. In a similar vein, Worthy et al. (2014) used model comparison to show differences in strategy use between younger and older adults in a decision-making task. Here, even though both age groups did not differ in overall task performance, older adults showed more evidence for a simple win-stay lose-shift heuristic compared to younger adults whose choices were best described by a weighted mixture of model-free and model-based RL.

### Model-based fMRI

In the field of cognitive neuroscience, there is a strong interest in model-based fMRI analyses that focus on neural correlates of latent model variables, such as reward prediction errors or state-action values (O'Doherty et al., 2007). For instance, a central finding in studies combining RL models with fMRI is the observation that the blood oxygen level-dependent (BOLD) response in the ventral striatum reflects a reward prediction error signal (e.g., O'Doherty et al., 2004; Delgado et al., 2008). Developmental neuroscientists thus examined whether age-related differences in the strength of neural signals associated with these variables can offer a mechanistic explanation for differences in behavior. Indeed, in older adults, the neural correlates of reward prediction errors seem to be impaired (Chowdhury et al., 2013; Eppinger et al., 2013a; Samanez-Larkin et al., 2014) but can be partially restored by a pharmacological intervention that increases intracerebral dopamine, leading also to enhanced task performance (Chowdhury et al., 2013). Thus, RL models can provide access to mechanisms (e.g., integrity of reward prediction error signal) that link the neural level (dopamine) and the behavioral level (task performance).

In adolescents, the evidence for alterations of the reward prediction error signal is less clear. A study by Cohen et al. (2010) reported an increased BOLD response to positive reward prediction errors in the ventral striatum for adolescents compared to both children and young adults, suggesting a mechanism for greater reward sensitivity during this age. However, other studies (e.g., van den Bos et al., 2012; Christakou et al., 2013) could not replicate this effect, possibly due to differences in task design or the participants' age range.

In most of the previous research in younger adults, the BOLD response in the vmPFC is associated with changes in state-action values during learning (Gläscher et al., 2009). Yet, so far there are only a few studies that looked at age-related changes in this signal. Christakou et al. (2013) report the neural representation of state-action values to become stronger from adolescence to adulthood; however, they did not find this to be related to behavioral differences. In older adults, state-action value signals are reduced (Tobia et al., 2016; de Boer et al., 2017) and signal strength predicts performance in a probabilistic decision-making task (de Boer et al., 2017), suggesting that the age-related deterioration of value signals in the vmPFC may explain the behavioral deficits.

To summarize, computational modeling can identify developmental differences in learning and decision-making not only by capturing quantitative differences in parameters that represent psychological processes but also by comparing qualitatively different formalizations of cognitive mechanisms and by detecting age-related changes in the neurophysiological implementation of these processes.

## Modeling social learning mechanisms across the lifespan

So far, we have only considered how individuals learn from their own actions. However, humans are fundamentally social beings (Fiske, 2009). For decades, psychologists have observed that social context influences decision-making and behavioral adaptation is realized in accordance with our social environment (e.g., Lewin, 1952; Asch, 1956). Yet most computational studies on the development of learning and decision-making have left social factors aside. The RL models reviewed above can be extended to describe mechanisms of social learning and exchange. In the following, we outline how such extensions have been implemented. We stress their relevance for key questions that have been asked about the development of social cognition over the past decades. We first tap into a process that remains important throughout the course of the human lifespan, namely the ability to learn *from* others, by observation, by social feedback, or from instruction. A further key question of developmental psychology has always been the development of social cognition or Theory of Mind (ToM; Frith and Frith, 2003). Thus, in a second step we describe how computational formulations can help to elucidate how we learn *about* others, that is, update our ToM about others.

### Learning from others

#### Observational learning

Although the process of learning from the consequences of one's own behavior through direct experience, as reviewed above, is pivotal for survival, it is rather time-consuming and potentially dangerous. Thus, many species have developed the ability to learn from others via observational learning (e.g., Tomasello et al., 1987), which is also of great interest from a human ontogenetic perspective as it is suggested as an important factor for cognitive and social development (Nielsen and Tomaselli, 2010; Meltzoff, 2013; Waismeyer and Meltzoff, 2017).

In RL terms, learning from observations means to compute observational prediction errors, namely the deviance of the expected reward and the reward that the other person receives, depending on the state the other person is in and the action the other person has undertaken.

(5) $$\begin{array}{c} \delta_{obs}=\underset{\text{observed reward}}{\underset{︸}{r_{obs}(s^{\prime })}}-\underset{\text{expected reward}}{\underset{︸}{Q\left(s,a\right)}} \end{array}$$

(6) $$\begin{array}{ccc} Q(s,a) & \leftarrow & Q(s,a)+\alpha_{obs}\delta_{obs} \end{array}$$

δ_*obs*_ is then multiplied by the observational learning rate α_*obs*_ which represents how fast participants learn from observed, not directly experienced outcomes. This update rule leads to an observationally-updated state-action value that individuals can use to make their own decisions. Observationally achieved state-action values can subsequently be updated using experienced prediction errors after taking an action and experiencing an outcome oneself.

Burke et al. (2010) applied such a computational account to a probabilistic, reward-based observational learning paradigm during fMRI in younger adults. They could show that observational outcome prediction errors correlated with activity in the vmPFC and the ventral striatum, similar to experienced outcome prediction errors (O'Doherty et al., 2004).

Learning from others' actions even in the absence of outcome information has been modeled using action prediction errors (Burke et al., 2010; Suzuki et al., 2012) that are computed as the probability that the observed choice *a*_*i*_ would not have been selected by oneself:

(7) $$\begin{array}{c} \delta_{act}=1-\underset{\text{expectation about action}}{\underset{︸}{P\left(a_{i}|s\right)}} \end{array}$$

Note that such action prediction errors are unsigned in nature, coding surprise about an observed action, rather than surprise and valence like in the case of a rewarding vs. punishing outcome. By means of this action prediction error, the choice probability is directly updated and the strength of this update is controlled by an imitation factor κ, in analogy to the learning rate in experiential learning (compare Equation 2).

(8) $$\begin{array}{c} P(a_{i}|s)\leftarrow P(a_{i}|s)+\kappa \delta_{act} \end{array}$$

Action prediction errors have been shown to be associated with activity in the dorsolateral prefrontal cortex in young adults (Burke et al., 2010; Suzuki et al., 2012).

A recent EEG study (Rodriguez Buritica et al., 2016) investigated observational learning in school-aged children by manipulating the amount of social information as well as the social partner the children were learning from (comparing child to adult learning model). The results of these study show that children seem to have problems to rapidly assess the informational value of social feedback during learning and consequently up-regulate their response to observed and experienced negative feedback, as reflected in the amplitude of medial prefrontal event-related potential (ERP) components. Moreover, children tend to imitate behavior more when the observed player is a child, compared to an adult, indicating that social information does impact the degree to which information is integrated during learning (Rodriguez Buritica et al., 2016).

Rodriguez Buritica et al. (2016) did not use computational modeling in their study. However, the computational account of observational learning described above could be readily applied to these data. One advantage would be that learning could be captured in a trial-by-trial manner (behaviorally as well as neurally), which avoids the block-wise average approach the authors have used here as an approximation to learning. In this framework, it would be interesting to contrast electrophysiological correlates of experiential and observed outcome prediction errors as well as action prediction errors in a modeling-informed trial-by-trial ERP analysis. Such modeling-informed single-trial analyses of the feedback-related negativity (FRN) and different types of prediction errors have recently been demonstrated in young adults (Ullsperger et al., 2014; Reiter et al., 2016). Given the relatively late maturation of the ventrolateral and particularly the dorsolateral prefrontal cortex (Gogtay et al., 2004) and the involvement of these brain regions in observational learning in young adults (Burke et al., 2010), it is apparent that an interesting next step would be to study the development of these processes using model-based fMRI, ideally in a longitudinal fashion. Imitation plays a crucial role for the acquisition of behavior from early infancy on (for example during language acquisition). The modeling account introduced here might prove useful to study the building blocks of imitative behavior, namely observed action prediction errors in toddlers. In the absence of choice data in early childhood studies, computational models could be fit to eye tracking data like saccadic response speed or pupillometry (Vossel et al., 2014; Hepach and Westermann, 2016) or electrodermal activity (Li et al., 2011b), using response models for continuous data.

Interestingly, an fMRI study in young adults has recently looked at the involvement of model-based processes in observational learning (Dunne et al., 2016). In this study, model-based observational prediction errors that are used to update one's internal model about the environment were associated with activation in the fronto-parietal network. Because model-based learning abilities change markedly over the course of one's lifespan (see above) and recent findings show that social cognition might age differently than non-social cognition (Reiter et al., 2017b), it would be intriguing to study model-based social learning processes from a (lifespan) developmental perspective.

#### Learning from social feedback

Humans are particularly prone to learning from social reinforcers, like a smile, praise, or a compliment and sensitive to learning from social punishment, like exclusion or rejection. From early childhood until later life, social feedback plays a crucial role during development and education in many areas, including language development or the development of social competences (Gros-Louis et al., 2006; Sebastian et al., 2010; Shinohara et al., 2012; Warlaumont et al., 2014). Translated to the RL modeling framework, this means that *r(s*′*)* for the computation of reward prediction errors (see Equation 1) can be social in nature. Indeed, neuroimaging studies have suggested a “common neural currency” for basic and social rewards (Behrens et al., 2008, 2009; Lin et al., 2011; Kishida and Montague, 2012) by demonstrating that social and monetary rewards elicit activation in overlapping brain regions.

In the developmental domain, one study (Jones et al., 2014) investigated learning from social feedback in children, adolescents and adults using a probabilistic learning task in combination with RL modeling and fMRI. In this study, different social cues were associated with different social reward probabilities. Social reward consisted of receiving a note from the co-player indicating interest in the participant. Surprisingly, the authors found a quadratic effect on learning rates for positive social feedback: Adolescents showed lower learning rates for positive feedback than children or adults. As discussed by the authors, this is in contrast to the common notion of higher sensitivity toward social reward in adolescents (Somerville, 2013; Foulkes and Blakemore, 2016). The authors argue that adolescents differentiate less between the cues that are associated with different amounts of positive social feedback.

Future studies interested in developmental differences regarding sensitivity to social feedback might more explicitly contrast learning from social reinforcers with learning from other (e.g., monetary) reinforcers across the lifespan. Computationally, fitting *r(s*′*)* as a free parameter (i.e., as a measure of reinforcement sensitivity; Gold et al., 2012), allows to compare sensitivity toward different types of reinforcers (e.g., social vs. monetary) between age groups. Recent modeling accounts have also captured subjective relevance in a Pavlovian conditioning approach (Katthagen, 2017). These computational approaches could be particularly suitable to re-assess the postulated higher relevance of social feedback in adolescence (Blakemore, 2008; Foulkes and Blakemore, 2016) using a modeling approach.

#### Learning from others' instructions

A human-specific ability pivotal for development and education is the ability to learn from others' instructions. For example, we do not need to burn our hands to learn not to touch a hot stove; a verbal warning from others is usually sufficient.

In RL modeling terms, instructions prior to one's own experience can be operationalized by changing the initial state-action value (i.e., in an experimental setting the state-action value of the first trial of an experiment) according to an instruction received before one's own experiences with this stimulus are gained. In the example above, this might mean that someone has actually told you how exceptionally tasty the chocolate ice cream from a certain ice cream parlor is. Instead of starting the ice cream tasting from scratch (i.e., in computational terms, with equal initial state-action values for all ice creams of 0, respectively), you might be biased toward white chocolate ice cream now (which might have a higher state-action value of, e.g., 0.8) before you have actually tasted it. To also model an ongoing effect of instructed knowledge *during* learning, an additional parameter can be introduced to the equation for updating state-action values which amplifies gains and reduces losses following the choice of the instructed stimulus (Doll et al., 2009).

(9) $$\begin{array}{c} Q\left(s,a_{instructed}\right)\leftarrow \{\begin{array}{c} Q\left(s,a_{instructed}\right)+\alpha_{instructed}\;\alpha_{+}\delta \;if\;\delta \geq 0\\ Q\left(s,a_{instructed}\right)+\frac{\alpha_{-}}{\alpha_{instructed}}\delta \;if\;\delta <0 \end{array} \end{array}$$

Here, α_*instructed*_ represents a parameter capturing instruction-biased updating. Neuroimaging studies in young adults have shown that instructions about rewarding outcomes modulate learning-related responses in the striatum (Doll et al., 2009; Li et al., 2011a) and vmPFC (Li et al., 2011a) and that this modulation might be dependent on the prefrontal cortex (Doll et al., 2009, 2011; Li et al., 2011a). This points toward the direction that learning from instructions builds upon the circuit that also supports learning through own experience.

So far, one study compared children, adolescents, and adults with respect to experiential reward learning vs. social instruction in a probabilistic reward learning task using computational RL modeling (Decker et al., 2015). While inaccurate instruction biased adults' estimations of a stimulus value, children and adolescents relied on their own experiences when estimating stimulus values through experience. These data suggest that when explicit instruction conflicts with experiential feedback about the value of an action, children, and adolescents weight their own experience more heavily. The prefrontal-striatal brain circuitry, which instruction learning builds upon, continues to mature into adulthood, which might serve as an explanation for these differences in learning between age groups.

Based on the reviewed developmental differences in instruction-based learning, it would be interesting to investigate how social feedback from different sources affects learning. In Decker et al. (2015), instructions were displayed on the screen without manipulating factors like the age (e.g., peer group vs. adult) or social distance (e.g., family member vs. friend vs. stranger) of the instructor. Such an experimental manipulation would allow fitting different bias parameters α_*instructed*_ for each social source condition, which could subsequently be compared between age groups. It should be noted, however, that manipulating social information in a laboratory can be very challenging, and the question arises whether the mere presentation of a face on a computer screen is sufficient to count as “social.” For future studies, it will be crucial to compare a laboratory situation to settings that are more naturalistic and under “real-life” constraints such as in school or kindergarten.

### Learning about others' mind

To understand when and how children develop their capacity to infer other people's mental states (ToM) has long been a “hot topic” in developmental psychology and has recently been extended toward research on lifespan development of social cognition (Henry et al., 2013; Reiter et al., 2017b). One influential idea concerning the implementation of ToM is that humans use and continuously update models for simulating and predicting others' behavior (Yoshida et al., 2008; Boorman et al., 2009; Diaconescu et al., 2014). One particular aspect of ToM is to infer the (potentially time-varying) motives of others during social interaction from their actions in order to determine their fidelity. Such social learning *about* others has recently also been translated into a computational model. In an experiment applied to young adults, participants were required to learn about the intentions of a confederate of whom they received advice to inform their next choice. The confederate's motivation to help or mislead (i.e., his fidelity) changed over time (Behrens et al., 2008; Diaconescu et al., 2014). In such a scenario, in computational terms, players would update an estimate of the confederate‘s fidelity, namely the probability of (un)faithful advice, according to the observed accuracy of the advice by concurrently tracking the congruency of advice and outcome. This idea could be incorporated into RL models; however Bayesian modeling approaches seem to be even better able to account for empirical data in this task (Diaconescu et al., 2014, 2017).

Applying the suggested modeling approaches for learning about other people's motives to developmental questions opens promising avenues for understanding the development of social cognition and social interaction. Tying together findings in young adults that social inference is influenced by uncertainty estimates, and findings from the non-social domain that uncertainty representation changes over the course of the lifespan (Nassar et al., 2016; van den Bos and Hertwig, 2017), it would be very interesting to investigate whether and how changes in uncertainty representation contribute to previously reported developmental differences in social cognition. Moreover, a recent study has demonstrated that distinct social prediction errors are associated with activation in different neuro-modulatory systems, respectively (Diaconescu et al., 2017): Lower-level prediction errors which updated predictions about an adviser's fidelity activated the dopaminergic midbrain, and genotypes favoring higher concentrations of dopamine were related to higher striatal activation associated with fidelity prediction errors. Higher-level prediction errors, updating the volatility of an adviser's intentions were associated with activation in the cholinergic basal forebrain. Notably, both neurotransmitter systems, dopamine, and acetylcholine, undergo marked changes over the course of the lifespan.

## Conclusion

In this review, we illustrated how researchers in the field of developmental cognitive neuroscience can make use of computational models to gain a more mechanistic understanding of lifespan differences in learning and decision-making. For both social and non-social settings, RL models provide a powerful technique to formalize the underlying mechanisms. Parameters that are derived from these models can be used to study developmental changes in learning and decision-making as well as the associated neural correlates.

We acknowledge that we are still in the early stages of this research. Some results seem to be inconsistent, possibly due to small sample sizes, differences in the employed paradigms or computational models. For instance, the studies investigating altered neural reward prediction error representations during adolescence (Cohen et al., 2010; van den Bos et al., 2012; Christakou et al., 2013) used different tasks (Iowa Gambling Task and a probabilistic learning task) with or without monetary rewards, and employed RL models that did or did not account for distinct learning rates after relative gains or losses. Future research should aim to identify the important boundary conditions of age-related effects. Furthermore, age-comparative fMRI studies tend to require many resources and therefore often do not involve large sample sizes, which complicates comparisons across studies. Moreover, to our knowledge all studies so far rely on cross-sectional designs, which limits the interpretability of the results. It would be desirable to fill this gap and to track the developmental trajectories of the computational underpinnings for learning and decision-making in a longitudinal manner.

In psychiatry, there is an increasing awareness that a computational understanding of mental illnesses is needed to improve clinical treatments (Montague et al., 2012; Huys et al., 2016; Reiter et al., 2017a). We believe that it is likewise necessary to comprehend the computational groundings of learning and decision-making during healthy development. This would allow us to create better learning environments in educational and occupational settings and adapt them to the specific needs of different age groups. Successful attempts along this direction have already been made. For example, in a study by Raufelder et al. (2016) learning rates and neural prediction error signals in a reversal learning task could be linked to different scholastic motivation types in adolescent pupils. We believe that several aspects of learning and decision-making discussed above also are of great practical relevance, such as learning from negative and positive feedback, the regulation of cognitive effort during (model-based) decision-making or the implications of learning from observations and instructions. Thus, knowing and understanding the cognitive processes involved in different types of learning and how they change with development might finally lead to advancements in lifelong education. Computational models constitute an essential part in this enterprise.

## Author contributions

FB, AR, and BE conceived the theoretical ideas; FB and AR conducted the literature review; FB, AR, and BE wrote the manuscript.

### Conflict of interest statement

The authors declare that the research was conducted in the absence of any commercial or financial relationships that could be construed as a potential conflict of interest.
